# A Clinical Update on Delirium: From Early Recognition to Effective Management

**DOI:** 10.1155/2011/875196

**Published:** 2011-06-16

**Authors:** Joaquim Cerejeira, Elizabeta B. Mukaetova-Ladinska

**Affiliations:** ^1^Serviço de Psiquiatria, Hospitais da Universidade de Coimbra, Praceta Mota Pinto, 3000 Coimbra, Portugal; ^2^Institute for Ageing and Health, Newcastle University, Newcastle upon Tyne NE4 5PL, UK

## Abstract

Delirium is a neuropsychiatric syndrome characterized by altered consciousness and attention with cognitive, emotional and behavioural symptoms. It is particularly frequent in elderly people with medical or surgical conditions and is associated with adverse outcomes. Predisposing factors render the subject more vulnerable to a congregation of precipitating factors which potentially affect brain function and induce an imbalance in all the major neurotransmitter systems. Early diagnosis of delirium is crucial to improve the prognosis of patients requiring the identification of subtle and fluctuating signs. Increased awareness of clinical staff, particularly nurses, and routine screening of cognitive function with standardized instruments, can be decisive to increase detection rates of delirium. General measures to prevent delirium include the implementation of protocols to systematically identify and minimize all risk factors present in a particular clinical setting. As soon as delirium is recognized, prompt removal of precipitating factors is warranted together with environmental changes and early mobilization of patients. Low doses of haloperidol or olanzapine can be used for brief periods, for the behavioural control of delirium. All of these measures are a part of the multicomponent strategy for prevention and treatment of delirium, in which the nursing care plays a vital role.

## 1. Introduction

Delirium is a neuropsychiatric syndrome of acute onset and fluctuating course, clinically characterised by altered level of consciousness, attention, and disturbance in orientation, memory, thought, and behaviour. The term delirium literally means, “out of the track”, and was firstly used by Celsus, in the first century A.D. to describe either states of agitation or excessive somnolence [1]. Historically, this syndrome has been described under different names and classifications [2]. Gradually the term delirium started to be more consistently used to designate reversible states of acute brain dysfunction, associated with fever or medical and/or surgical conditions.

Delirium is a common occurrence in medical or surgical wards and affects particularly elderly people with comorbidities and prior cognitive impairment. Thus, this syndrome affects 11–42% of medically ill patients [3] and complicates 24–89% of hospitalizations for elderly patients with dementia [4]. The prevalent rates in the community are somewhat lower, ranging from 0.5% [5] to 13% for older adults with dementia [6]. In elective orthopaedic surgery, the incidence of postsurgical delirium is between 9–28% [7, 8]. Higher rates are seen in emergent hip fracture surgery, in which a large proportion of patients present with preoperative (4%–36%) or postoperative delirium (up to 53%) [9]. Postoperative delirium after cardiac surgery varies from 2 to 57%, according to the procedure, type of patients and methodology of the study [10–13]. In the context of sepsis, delirium affects, 9% to 71% of patients [14].

Delirium is independently associated with adverse outcomes consisting not only of increased hospital stay, morbidity and mortality, but also long-term effects such as cognitive and functional deterioration and higher rates of institutionalization [15–19]. Delirium, therefore, imposes a significant economic burden on health care systems, standing side by side with diabetes mellitus and falls as a major cause of increasing costs being directly responsible for an additional cost of more than $60,000 patient/year [20]. Despite its importance, health care professionals often fail to recognise the syndrome, and acute medical care has been found insufficient to meet the complex care needs of these frail older adult. Also, more specialised clinical services, for example, acute care for the elderly, have been associated with a decreased incidence of delirium [21], suggesting that the multidisciplinary approaches may be more beneficial in both detecting and treating delirium.

The clinical relevance of delirium has led to the development of best practice guidelines by the American Psychiatric Association, [22, 23] British Geriatrics Society (BGS) [24], and, more recently, by the National Institute of Clinical Excellency (NICE) [25] which is a particularly exhaustive and cutting-edge document. Since the publication of the NICE guidelines, a number of additional pharmacological prophylaxis and treatment studies have followed and have contributed to further development of our understanding of best practice for delirium.

Nurses are in a particularly relevant position in the healthcare system to improve detection rates, manage and provide necessary care to people with delirium, and prevent these episodes in those at high risk [26]. In the current paper, we reflect on the clinical recognition and early diagnosis of delirium, as well as the available evidence addressing the most effective measures for prevention and treatment.

## 2. Clinical Definition and Psychopathological Features of Delirium

Health care staff, including nurses, should be aware that different terms used in scientific literature and in clinical practice (e.g., acute confusional state, encephalopathy, acute brain failure, organic brain syndrome) all refer to conditions that would meet the definition criteria of delirium. At present, delirium is included in the two main classification systems: the revised fourth edition of the Diagnostic and Statistical Manual of Mental Disorders (DSM-IV-TR) [27] and the International Classification of Diseases (ICD-10) [28].

**DSM-IV-TR Diagnostic Criteria**

In DMS-IV-TR delirium is defined by the presence of disturbed consciousness (i.e., reduced clarity of awareness of the environment with reduced ability to focus, to sustain, or to shift attention) and a change in cognition (such as memory deficit, disorientation, or language disturbance) or the development of a perceptual disturbance that is not better accounted for by a preexisting, established, or evolving dementia. The disturbance develops over a short period of time (usually hours to days) and tends to fluctuate during the course of the day. There should be evidence from clinical history, physical examination, and/or laboratory findings, that the disturbance is caused by direct physiological consequences of either a general medical condition, substance intoxication/withdrawal, or multiple etiologies [27].

**ICD-10 Diagnostic Criteria**

The diagnostic criteria for delirium provided by the ICD-10 require the presence of five clinical features of the syndrome: impaired consciousness and attention, global disturbance of cognition as well as psychomotor, sleep and emotional disturbances [28]. This can probably explain why ICD-10 has relatively lower sensitivity than DSM-IV [29].

### 2.1. Core Symptoms of Delirium

The fundamental psychopathological features of delirium are disturbed consciousness (level of awareness or the ability to stay awake) and reduced levels of attention (capacity to recruit and maintain senses focused on relevant, stimuli) which directly impair the capacity to monitor, select and integrate cognitive stimuli.

Inattention is the most frequent clinical finding in a delirium episode. If severe enough, it can be detected during a clinical interview (e.g., the patient is unable to follow a conversation). In mild cases, impairment of attention is elicited only by formal cognitive testing (e.g., digit span, serial sevens, or naming the months in reverse order). Attention is impaired in early stages and throughout the course of a delirium episode, correlating with the severity of cognitive deficits [30, 31].

Consciousness refers to the state of being awake and able to interact with the environment, allowing the integration of stimuli within the cognitive experience. In this sense, disturbed consciousness can be considered as an impairment in alertness (wakefulness), awareness, and arousal [32]. When present in a patient with an acute medical condition, the likelihood of delirium is high [29].

The sudden and global impairment in cognition characteristic of delirium is manifested as difficulty in

- orientation (impaired awareness of oneself and one's surroundings in terms of time, place and person);
- memory (impaired ability to learn new information or to recall previously learned information);
- language and thought (disturbance in the comprehension and/or expression of speech as well as abnormalities in the flow and connectivity of thought);
- visuospatial abilities (impaired capacity to construct and draw geometric configurations).

Specific deficits in visual perception, not necessarily related with cognitive performance, have been described in people with delirium [33]. These may underlie perceptual disturbances, particularly of visual modality, such as

**Figure 1:**
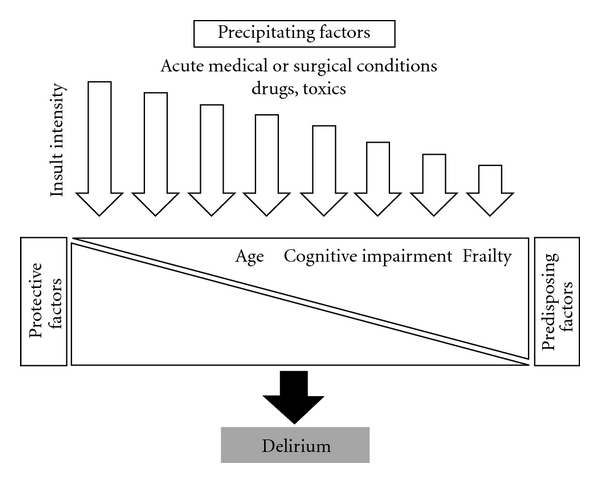
Relationship between predisposing, protective, and precipitating factors in delirium.

- illusions (misinterpretations of real sensory stimuli, as when the patient in a dark environment sees a threatening figure emanating from shadows on the walls) (see Figure 1),
- hallucinations (ranging from simple flashes or unstructured sounds to elaborate visions, that occur without corresponding sensory stimuli).

### 2.2. Associated Symptoms of Delirium

A range of behavioural and emotional symptoms, not specifically described in DSM-IV criteria but more adequately reflected in ICD-10, are frequently observed during delirium: sleep-wake disturbances, lability of affect, delusions, and motor disturbances.

*Disturbances in sleep-wake cycle* are common in patients with delirium ranging from mild sleep continuity disturbance at night or occasional drowsiness during the day to severe circadian fragmentation with multiple periods of sleep and wakefulness.

When present, *delusional ideas* consist of false beliefs usually of suspicious or persecutory content (paranoid ideation) poorly systematized and containing relatively few elements (simple delusions). Patients often do not spontaneously verbalize these ideas as they are frightened and quite guarded. Instead, they are more likely to manifest a range of emotions associated with a sense of threat (apprehension, worry, irritability, or “distress” as stated in NICE guidelines [25]). Not surprisingly, *lability of affect* (or mood) is frequently observed in subjects with delirium and this is characterized by rapid emotional shifts, often within seconds to minutes. Contrary to common belief, there is evidence that most patients will retain in the future, vivid and detailed memories about experiencing delirium. Acute confusion is remembered as a state dominated by mixed emotions associated with recent and distant memories of events, places, and people with dissolution of time and space which are organized in a dream-like narrative. Although this can sometimes be difficult to express in words, for most patients delirium is a negative experience, inducing discomfort even after recovery, originated by worries about what had happened to them [34].

Changes in *motor behaviour* have been recognized for long in patients with delirium involving either overactivity (e.g., restlessness or agitation) or underactivity (motor retardation). Aggressive behaviour may occur as a consequence of increasing levels of paranoid ideation and irritability, potentiated by other factors (e.g., hunger, sleepiness, and pain). Trying to reassure the patient often increases suspiciousness as the interviewer may be perceived as being involved in the plot. Thus, it is usually more helpful to acknowledge the patient's concerns and to follow a patient-centred interview in order to trace his beliefs.

Overall, there is a lack of evidence about the different patterns of motor activity and their stability during a delirium episode. Particularly, there is a lack of agreement about whether motor features should be independently assessed or clustered with the associated neurobehavioural symptoms [35]. 

### 2.3. Delirium Subtypes

In clinical practice, it is common to classify delirium into different subtypes, based on the predominance of these “psychomotor features”. Thus, the hypoactive subtype is characterized by reduced alertness, sedation, and reduction of motor activity, whereas, the hyperactive form is associated with hypervigilance, overt psychotic features (e.g., hallucinations, delusions), and agitation. Please note that psychotic features are also present albeit less frequently in people with hypoactive delirium (reviewed by Friedlander et al. 2004 [36]). As it became apparent that most cases of delirium have overlapping features of these two subtypes, a third, mixed, subtype was proposed [37]. However, in recent years, it has been noted that a small portion of delirium patients (3–22%) do not have evidence of a motor component in their clinical symptomatology over the previous 24 hours, and thus do not meet the criteria for hypo-, hyper-, or mixed form of delirium [29, 38, 39]. In this context, a new subtyping scheme proposed by Meagher et al., designed for use by medical and nonmedical professionals, can be a useful tool to characterize the motor features of delirium [39].

Interestingly, the hyperactive subtype is still considered as the predominant form of delirium (up to 80% of medical students, Mukaetova-Ladinska, unpublished data), despite the lower prevalence rates. In fact, the mixed and hypoactive subtypes are more prevalent (55% and 46%, resp., [40, 41]). In particular, the hypoactive delirium type is associated with higher mortality [42], older age (22% in adults versus 41% in elderly [40]), palliative care needs [43], polycomorbidity [44], and severity of illness [45] and is usually either overlooked and undiagnosed (in particular in intensive care setting [41]) or misdiagnosed as depression or fatigue [43]. Thus the current prevalence rates for the hypoactive delirium may be largely underestimated. 

## 3. Pathophysiology of Delirium 

Delirium is the clinical manifestation of an acute and global disruption in brain homeostasis, resulting in the failure of high integrative cognitive, behavioural, and emotional functions (discussed above). Thus, any kind of insult affecting the neurophysiological processes of the central nervous system (CNS) can elicit an episode of delirium.

### 3.1. The Multifactorial Nature of Delirium

In the light of a diversity of medical or surgical conditions associated with delirium, it remains largely unknown how pathophysiological changes occurring in the periphery can result in the disruption of brain function. Yet, it is clear that some factors (e.g., increased age, dementia, high burden of comorbidities) render the subject more prone to develop delirium when exposed to even minor insults (e.g., urinary tract infection). Also, numerous studies in different clinical settings have shown that delirium is a heterogeneous condition and can be elicited by the combined action of a diversity of factors (Table 1). Thus, the pathophysiology of delirium is currently conceptualized as being the complex and dynamic interplay between predisposing, protecting, and precipitating factors in a given patient. Most common precipitating factors of delirium are medication, metabolic disorders, infection, surgical procedures, and CNS disorders (Table 2).

### 3.2. Proposed Pathophysiological Pathways

Several studies which have focused on identifying the neurochemical changes during delirium found an imbalance in all the main neurotransmitters (acetylcholine, serotonin, dopamine, glutamate, GABA). Also, neuropeptides, catecolamines, cortisol, and inflammatory markers have been implicated in delirium pathophysiology [48]. 

Currently, the two main theories for delirium pathophysiology are cholinergic deficiency and aberrant stress response/neuroinflammation.

Probably, the first evidence supporting the cholinergic deficiency hypothesis originated from the observation that delirium, cognitive impairment, and psychosis are induced by toxics (*Atropa belladonna*) and drugs with anticholinergic action (e.g., tricyclic antidepressants, antihistamines) [49]. In addition to direct pharmacological antagonism, failure of cholinergic system during delirium has been proposed to be also the result of disruption in acetylcholine (ACh) synthesis, transport, and release [50]. Indeed, metabolism of ACh is intimately related to energetic status of neurons as it of its steps are dependent on adenosine triphosphate (ATP, the major source of energy for most metabolic processes in living organisms and cells) and its precursor Acetyl-Coenzyme A. Any insult that affects the oxidative chain, such as hypoxia or inflammation, can then impair ACh availability in the brain and disrupt several cognitive processes. Acetylcholine deficit, due to cholinergic neuronal loss, has been considered a potential mechanism explaining the recognized susceptibility of patients with dementia to develop delirium [51]. Thus, despite being elicited by a wide number of causes, central cholinergic deficit has been proposed as a “final pathway” to delirium considering that its clinical presentation is relatively stereotyped [52, 53].

More recently, animal experiments, particularly those by Cunningham et al., have clearly shown that peripheral or local precipitating factors (e.g., lipopolysaccharide) can evoke pathophysiological events within the Central Nervous System (CNS), with production of proinflammatory cytokines by microglial cells. These neuroinflammatory changes are coupled with associated cognitive and behavioural disturbances, the so-called “sickness behavioural syndrome” similar to delirium [54, 55]. A related hypothesis postulates that an aberrant stress response with exaggerated production of cortisol underlies the pathophysiological features of delirium [56]. In line with this evidence, several studies in medical and surgical patients have shown that plasma levels of several inflammatory markers, particularly IL-6, and cortisol are altered before and/or during delirium supporting the aberrant stress response/neuroinflammatory hypothesis of delirium. Interestingly, a significant interaction exists between the cholinergic system and the innate immune response through the “cholinergic anti-inflammatory pathway” [57]. Although not presently available, pharmacological strategies that can modulate the aberrant stress response/neuroinflammatory pathway may well offer new therapeutic tools to be used in the management of delirium.

**Neurochemical and Neuroimaging Changes in Delirium**

Functional neuroimaging studies report significant although nonspecific reductions in brain blood perfusion [58] and white matter hyperintensities [59] during delirium. Similarly, lower fractional anisotropy values (determined by diffusion tensor imaging) of deep white matter and thalamus have been identified in elderly with postoperative delirium [60]. However, since many of the delirious patients in these studies had impairments in memory, executive function, and attention at three months, in absence of baseline cognitive impairment [59], further studies are now needed to examine the relationship between currently available imaging techniques and their usefulness in diagnosing and further characterisation of the delirium syndrome. Since dementia with Lewy bodies and delirium share somewhat similar clinical phenotype [61], it would be of interest to see whether the currently available DaTSCAN (Ioflupane, 123-I FP-CIT) may find a role in the differential diagnosis of delirium.

## 4. Delirium Diagnosis

Considering that delirium has no pathognomonic features, central to the diagnosis is the identification of a cluster of nonspecific signs and symptoms (described above) within a temporal frame that links the onset or exacerbation of a general medical condition (and/or substance use) to the change in mental status. Thus, there is an overall agreement that delirium is far more frequent than it is recognized by medical or nursing staff. Importantly, under or misrecognition of delirium is associated with adverse outcomes, including increased mortality [62]. The first step to address this problem is to increase the awareness of all healthcare providers, even nonspecialists, about the clinical relevance of delirium in clinical setting [25].

### 4.1. Recognizing the Warning Signs of Delirium

A high degree of clinical expertise is crucial to detect any acute change in patient's mental status presenting as the early signs of delirium. Recognition of hypoactive delirium is particularly challenging and demands a careful monitoring of patient's behaviour at the bedside in order to detect worsened concentration, reduced mobility or motor activity, changes in appetite or social withdrawal.

The presence of factors associated with increased risk of delirium (age ≥ 65, prior or present cognitive impairment, current hip fracture, and severe illness) warrants a more close clinical monitoring. Irrespective of that, all patients admitted to the hospital should be regularly assessed for delirium (at least daily) [25]. Some authors recommend daily chart reviews of patient status based on nursing and medical notes as a complementary method to detect delirium. Although this can be a source of misidentification of delirium (especially when the patient has dementia, severe illness or high baseline delirium risk [63]), chart-based detection associated with standard assessment has proved to be useful in some particular settings, such as in Intensive Care Units [64].

Use of predictive models can also be a cost-effective method of selecting patients with high-risk of delirium, in whom screening should be done as a routine practice. The distinction between the longstanding cognitive impairment and the acute onset confusion relays on a good collateral information from relatives and carers. A simple question directed to a patient's friend or relative (“Do you feel that [patient's name] has been more confused lately?”) has shown to have a sensitivity of 80%, a specificity of 71%, and a high negative predictive value (91%) in relation to the diagnosis of delirium based on a psychiatric interview [65]. When assessing patients with delirium, it is necessary to use a more focused and structured interview when compared to other patients. This can be achieved by using simple, closed-ended questions, redirecting the patient and do not allow long periods of silence.

### 4.2. Confirmation of Diagnosis

A healthcare professional proficient in the diagnosis of delirium should carry out the assessment of patients presenting with warning signs. Although DMS-IV or ICD-10 criteria remain the gold-standard for diagnosis of delirium, Confusion Assessment Method (CAM) and CAM-ICU (for critically ill patients) are valid alternatives [25].

The CAM is a simple instrument initially developed for screening of delirium by trained nonpsychiatrists. The diagnostic algorithm, based on DSM-III-R criteria, assesses four features: (1) acute onset and fluctuating course (2) inattention (3) disorganized thinking (4) altered level of consciousness. Delirium is diagnosed when both acute onset/fluctuating course and inattention are present (features 1 and 2) and at least one of other two features. CAM was first validated in a sample of 56 medical patients and found to have high sensitivity (94 to 100%) specificity (90–95%) and inter-rater reliability when compared to psychiatric comprehensive assessment [66]. It is easy to administer and can be completed in 5–10 minutes based on observations made during an interview that should include some formal cognitive assessment (e.g., Mini-Mental State Examination). In hospital setting, the CAM test has a sensitivity ranging from 43% to 90% and a specificity from 84% to 100% (when using DSM-IV criteria as the reference) [67]. The CAM-ICU assess on the presence or absence of the following features: acute onset or fluctuation course and inattention and either disorganized thinking or altered level of consciousness, and has a sensitivity to detect delirium of 93–100%, and specificity of 98–100%, and similarly, high inter-rater reliability [68]. When the CAM is rated by untrained nurses and without any formal cognitive assessment, delirium is often unrecognized [69]. Thus, although CAM is currently the most widely used instrument for detection of delirium, adequate training is necessary to enhance its sensitivity and specificity. Furthermore, this training should involve nursing staff engaged in the care of people with delirium, to improve their clinical skills in detecting and monitoring these patients.

### 4.3. Additional Assessment

Apart from CAM, a large number of other instruments have been developed to improve detection rates of delirium and/or to measure its severity (reviewed in Adamis et al. [70]). Among those, some have been adequately validated.

- Delirium Rating Scale (DRS) was designed to measure the severity of delirium [71]. A substantially revised version of DRS was published in 1998 (DRS-R-98), consisting of a 3-item diagnostic section and 13-item severity section to be scored in the first assessment. For longitudinal assessments only, the severity items should be rated from 0 to 3 points based on all sources of information available [72].
- Memorial Delirium Assessment Scale (MDAS) has been widely used in critical care and in patients with advanced cancer. It consists of a 10-item scale assessing for cognition (3 items) and neuropsychiatric symptoms and is best suited for quantification of delirium severity rather than for screening or diagnosis [73].
- The Neelon and Champagne (NEECHAM) Confusion Scale was developed by nurses in order to assess patients based on observations in the course of providing care to patients. It contains nine items divided into three subscales (responsiveness-information processing, behavior, and vital functions). [74] A score of 30 indicates normal function and 0 severe confusion with a cutoff point for delirium of 24.
- Delirium Observation Screening Scale (DOS) was developed to rate nurses' observations during regular care [75]. The scale has a good predictive validity against the diagnosis made by a geriatrician based on DSM-IV criteria and has a concurrent validity, as tested by comparison with CAM, of 0.63.

Nonspecific tests for delirium (e.g., MMSE [76], Clock drawing test [77]) can also be useful for establishing the baseline cognitive level of the patients, which can be compared with the subsequent assessments. However, they are not recommended as screening or diagnostic tools for delirium [25].

### 4.4. Differential Diagnosis with Dementia

DSM-IV-TR underscores the importance of considering a pre-existing, established or evolving dementia that can better explain the symptoms of delirium. Yet, there is a close association between the two conditions. Thus, delirium complicates 24 to 89% of inpatient stays for elderly patients with dementia [4]. Inversely, the available evidence strongly suggests that delirium increases the risk of new-onset dementia at long term, as much as 6-fold at three-year followup [17]. Also, people with pre-existing dementia suffer from an acceleration of cognitive decline following an episode of delirium [78].

Delirium is said to be superimposed on a dementia when an acute change in mental status occurs in a subject with an ongoing dementia. Failure to differentiate delirium from pre-existing dementia is clinically relevant as it can lead to serious medical conditions being missed and thus not treated. This may be particularly true when behavioural or cognitive changes in a patient with dementia are attributed to “normal” fluctuation of symptoms of the underlying dementia than to superimposed delirium [79].

While both delirium and dementia are characterized by a global impairment of cognition, they can be differentiated based on clinical features and natural course (Table 3). Thus, in delirium the global cognitive impairment emerges rapidly in a patient with disturbed consciousness and attention in the context of a medical or surgical condition. This contrasts with demented nondelirious subjects in whom cognitive impairments are primarily caused by the sustained, progressive brain disorder rather than by a dysfunction of consciousness and attention which are generally preserved. However, there is a substantial clinical overlap between the two conditions and it may prove difficult to differentiate between the 2 syndromes. 

There is no strong evidence that delirium has distinct features when occurring in people with prior dementia [80]. However, recent studies suggest that level of consciousness and hyperactive motor features can be more frequent among delirious demented patients than in nondemented patients with delirium [81, 82]. The key issue in diagnosing delirium in demented patients is to determine whether there is a change in the baseline clinical picture or whether the presenting symptoms are only an expression of pre-existing cognitive disorder. For that, it is crucial to have knowledge of the premorbid mental status, which can be achieved by clinical evaluation and/or collateral information (family caregivers, family practitioners, etc.). Clinical diagnosis of delirium in patients with dementia should focus, therefore, primarily on assessment of level of consciousness and attention rather than global cognitive impairment, which are common to both. Nevertheless, the crucial issue in the clinical daily practice is not so much to classify a patient as having pure forms of delirium or dementia but, rather to identify and remove the reversible components of the clinical picture [83]. Thus, in practical terms any confused patient should be considered to have delirium until proven otherwise.

### 4.5. Factors Associated with Delirium Under Recognition

The hypoactive form of delirium (representing the majority of cases, as reviewed above) and delirium in individuals with advanced age, sensorial deficits, prior cognitive impairment, or dementia and medical problems like infection or dehydration are the main reasons for the acute confusional sate not being recognised and diagnosed [69, 84, 85]. Hypoactive delirium and dementia comorbidity, appear to be the best predictors for overlooking delirium in the elderly with cognitive impairment [69]. Similarly, presence of dementia influences the survival time and is associated with higher death risk, irrespectively of the delirium subtype [86, 87]. This may be due to the higher rates of polycomorbidities [44] and use of neuroleptic medication in dementia subjects, already described to be associated with higher death risk [88]. All these findings indicate the need for further improvement of the medical status and care, especially for the elderly with dementia, in order to decrease the risk factors for a delirium episode in this frail population. Additional causes of underrecognition of delirium associated with healthcare providers include misinterpreting compliant behaviour as an indicator of intact cognition and normalization of behaviour changes (e.g., “he's only tired”, “it is normal to get confused during an infection”).

## 5. General Assessment

A detailed medical history, preferably obtained from different sources, should assess comorbidities and must include a careful review of medication with particular attention to recently introduced or discontinued drugs and those with anticholinergic potential. Physical examination should evaluate major systems and vital signs searching for medical causes of delirium. Pain and sensorial impairment should also be assessed. Useful auxiliary exams generally include blood count, electrolytes, renal, hepatic function, urine analysis, chest X-ray and ECG. Whenever considered necessary, other assessments should be requested, for a complete evaluation (e.g., toxicologic analysis, neuroimaging, lumbar puncture) (Table 4). Frequently, patients with dementia simultaneously have multiple subthreshold conditions whose conjugation can precipitate delirium (e.g., pressure ulcers, urinary retention, faecal impaction, dehydration). So, although no sole predominant cause can be identified, all the potentially relevant precipitating factors should be corrected as possible.

## 6. Management of Delirium: Prevention and Treatment

### 6.1. Medication Review

Once delirium is recognized in a patient (with or without dementia), a prompt and thorough clinical and laboratorial evaluation should be made to identify precipitating causes, which must be corrected as soon as possible. Drug review is one of them, in order to minimize the potential risk factors contributing to a delirium episode. It is essential to review urgently the medications, stop any non-essential drugs, and those with significant anticholinergic effect as well as address possible drug interactions (reviewed in Alagiakrishnan and Wiens 2004 [89]). A recent systemic review [90] highlighted several groups of drugs that increase the risk for delirium, such as opioids (OR 2.5, 95% CI 1.2–52), benzodiazepines (OR-3.0, 95% CI 1.3–6.8) dihydropyrdines (OR = 2.4, 95% CI 1.0–5.8), and antihistamines (OR = 1.8, 95% CI 0.7–4.5), but not neuroleptics (OR-0.9, 95% CI 0.6–1.3) or digoxin (OR = 0.5, 95% CI 0.3–0.9). The findings from this review recommend considering reducing and/or stopping benzodiazepines in subjects who are already on them and avoid further new prescriptions of this group of drugs, whereas the opioids should be prescribed with caution, in people with unregulated pain. 

### 6.2. Nonpharmacological Interventions

Multicomponent interventions targeting specific risk factors for delirium have been developed through training and educational programmes of health care staff, nonpharmacological intervention protocols and improvement of the environment of the patient [91]. Globally, these interventions, dependent on the provision of high-quality nursing care, are effective in reducing the incidence, severity, and duration of delirium.

Multicomponent interventions have been shown to be cost-effective in preventing delirium when applied to patients at risk of delirium in a hospital setting. Important risk factors  do  address and  include  cognitive  impairment/disorientation, dehydration/constipation, hypoxia, infection, immobility, pain, medication, nutrition, sensory impairment, and sleep [25].

These nonpharmacological interventions should be also offered to every patient with delirium and include promoting day activity, maintaining quite, well-lit environment, staff continuity, avoiding room and bed changes, providing hearing and visual aids, encouraging personal items, limiting visits especially for hyperactive delirium patients, remove noxious stimuli (e.g., catheters, pumps, etc.), limiting medical monitoring and testing (e.g., measuring blood pressure, temperature, blood works) [25]. A recent study on elderly with dementia also found improvement of severity and duration of delirium in those elderly randomized to cognitively stimulating activities [92]. 

### 6.3. Pharmacological Measures

Preventive pharmacological studies have been published with different classes of medication (typical antipsychotics, atypical antipsychotics, benzodiazepines, cholinesterase inhibitors). Two placebo-controlled trials reported a reduced incidence of delirium following administration of risperidone [93] and olanzapine [94] perioperatively in cardiac and orthopaedic surgery. Despite this promising results, the current available evidence is insufficient to recommend a pharmacological strategy for delirium prevention.

The use of medication is not a first-line strategy in the treatment of a patient with delirium [25]. In some distressed patients with hyperactive symptoms, such as agitation or hallucinations, haloperidol or olanzapine can be used cautiously (lowest effective dose for less than 1 week) [25]. Risperidone (0.5–1 mg) and quetiapine (25–50 mg) are reasonable alternatives [95]. Benzodiazepines are the mainstay pharmacological treatment for delirium associated with alcohol withdrawal (*delirium tremens*) but not for other causes.

Although a number of case report studies have found benefits of use of cholinesterase inhibitors (donepezil and rivastigmine) in the treatment [96–99] and prevention [98, 100] of delirium, randomized control trials have failed to provide evidence for the effectiveness of either donepezil [101] or rivastigmine [102, 103] in the treatment or prevention [104] of delirium. Furthermore, the most recent randomized control study in critically ill patients had to be halted, due to the higher mortality rate in the rivastigmine group compared to the control subjects (*P* = .07). Furthermore, rivastigmine was associated with a more severe type of delirium, that is, a longer stay in intensive care unit. [103] The reported differences between the outcomes in case reports and the open labelled trial [105] and those in the randomized control trials may be attribuTable to the severity of medical illness, heterogeneous clinical samples and extent of polypharmacy. Similarly, further work would be required to identify whether the effectiveness of cholinesterase inhibitors may be confined to a distinct subgroup of people with delirium, for example, elderly with cognitive impairment.

## 7. Conclusions

As reviewed above, delirium is a neuropsychiatric syndrome commonly observed in hospital setting being associated with a wide range of adverse outcomes. The nursing care the delirium patients receive, as well as the nursing role within the multidisciplinary delivery of care is of upmost importance. The NICE guidelines for delirium (2010) clearly identify the important role of nursing staff not only in providing patient-centred care (taking into account patients' needs and preferences, facilitating good communication), but also in undertaking adequate interventions to prevent delirium (management of environment, maintaining a familiar team of healthcare professionals), and delivering of nonpharmacological interventions (ensuring effective communication and reorientation, providing reassurance, engaging family, friends and carers, so that stress and violence be avoided or reduced/minimised). Although the current NICE guidelines for delirium do not mention the involvement of liaison team members, the multidisciplinary involvement should include the expertise of Liaison Old Age Psychiatry teams [106], that will enhance the delivery of effective nursing care, and facilitate educational approaches [107] for dealing with the acutely medically confused elderly. 

## Figures and Tables

**Table 1:** Risk factors for delirium [10, 46, 47].

(A) *Medical setting *	
Visual impairment	Severe illness
Cognitive impairment	Dehydration

(B) *Postoperative delirium *(cardiac surgery)	

Cerebrovascular disease	Diabetes mellitus
Peripheral vascular disease	Preoperative atrial fibrillation
Impaired left ventricular ejection fraction	Preoperative cardiogenic shock
Urgent operation	Intraoperative haemofiltration
Prolonged duration of surgery	High blood transfusion requirement

(C) *Postoperative delirium *(non-cardiac surgery)	

Cognitive impairment	Older age
Functional impairment	Sensory impairment
Depression	Preoperative psychotropic drug use
Psychopathological symptoms	Medical comorbidity

**Table 2:** Conditions commonly associated with delirium.

*Primary CNS disorders*	
Traumatic brain injury	Abscess
Stroke	Subdural haematoma
Tumours	Encephalitis
Seizures	

*Systemic disorders*	
Inflammatory/infectious	
Sepsis	Pneumonia
Trauma	Urinary tract infection
Organ dysfunction	
Electrolyte abnormalities	Hypoglycaemia, Hyperglycaemia
Renal failure	Hepatic failure
Neoplasm	Burns
Cardiac insufficiency	Respiratory insufficiency
Anaemia	Pain

*Substance-induced*	
Medications	
Anticholinergic	Antibiotics
Opioids	Anaesthetics
Sedative-hypnotics	Antineoplastics
Corticosteroids	Antihypertensives
Drugs of abuse	Toxics

**Table 3:** Differentiating Delirium from Dementia.

Features	Delirium	Dementia
Onset	Clear-cut, acute (hours to days)	Insidious (months to years)
Identifiable precipitant	Yes	No
Course	Fluctuating (sun-downing effect)	Stability of symptoms within days
Duration	Reversible Resolution in days or weeks	Not reversible Continuously progressive
Level of consciousness	Impaired	Usually not impaired (exception: DLB, VaD)
Level of attention	Impaired	Usually not impaired (exceptions: DLB, VaD, FTD)
Mood changes	Frequent	Rare (exceptions VaD)
Hallucinations, Illusions	Frequent, predominantly visual	Rare (exception: DLB)
Delusions	Frequent (fluctuating, fragmented)	Rare
Motor activity	Hyperactive/Hypoactive/Mixed	Without specific features

DLB: Dementia with Lewy Bodies

VaD: Vascular Dementia

FTD: Fronto-Temporal Dementia.

**Table 4:** General assessment of confused patient to identify and treat possible causes.

Physical frailty	Sensory impairment (deafness, visual)
Severe illness	Surgery (e.g., fractured neck of femur)
Dementia	Alcohol withdrawal
Infection	Renal impairment (electrolytes imbalance)
Dehydration	Neurological deficit (e.g., stroke, epilepsy)
Constipation	Glycemic control
Medication (drug toxicity,	Pain
polypharmacy, side effects)	
